# The Microscope

**Published:** 1883-05

**Authors:** C. H. Stonewall

**Affiliations:** Ann Arbor, Mich.


### The "Microscope."

The first number of the third volume of this valuable journal is at hand. Two and a half years ago, Prof. Stowell entered upon its publication with considerable misgiving as to success, being in doubt as to whether the subject of microscopy had become of sufficient interest in the west to warrant such an undertaking.

But its success has proved that his fears were unfounded; its circulation has steadily increased from the begining, until it is now a paying institution. It has been enlarged from time to time, and now contains forty-eight pages exclusive of the advertisments.

Its matter is improved—now very good indeed—always was good—there is now a large number of contributors, and more material from which to select. Every number contains more or less of illustration.

For some things the *Microscope* is certainly to be commended. It is eminently practical, and yet deals with principles all the while, it contains practical suggestions and directions, valuable to every microscopist. Its pages are not running over with egotism and self-conceit. It does not claim to be the leading microscopic journal of the world or of this country.

During Prof. Stowell's illness, Mrs. S. edited and issued the January number, which was one of the best of the series. No one who knows Mrs. S., however, would be surprised at that.

There is a growing interest in the dental profession on the subject of microscopy, and I have no hesitancy in saying, that the members of the profession can no where else so well and so economically supply themselves with a periodical on this subject.

Those who wish this journal should address Prof. C. H. Stowell, Ann Arbor, Mich.

